# Tuning the Optical and Electrical Properties of ALD-Grown ZnO Films by Germanium Doping

**DOI:** 10.3390/ma17122906

**Published:** 2024-06-14

**Authors:** Sylvester Sahayaraj, Rafał Knura, Katarzyna Skibińska, Zbigniew Starowicz, Wojciech Bulowski, Katarzyna Gawlińska-Nęcek, Piotr Panek, Marek Wojnicki, Sylwester Iwanek, Łukasz Majchrowicz, Robert Piotr Socha

**Affiliations:** 1 CBRTP SA Research and Development Center of Technology for Industry, Ludwika Waryńskiego 3A, 00-645 Warszawa, Poland; sylvester.sahayaraj316@gmail.com (S.S.); rafal.knura@cbrtp.pl (R.K.); kat.skibinska01@gmail.com (K.S.); wojciech.bulowski@cbrtp.pl (W.B.); or marekw@agh.edu.pl (M.W.); sylwester.iwanek@cbrtp.pl (S.I.); 2 Institute of Metallurgy and Materials Science, Polish Academy of Sciences, 25 Reymonta, 30-059 Kraków, Poland; starowicz.z@imim.pl (Z.S.); gawlinska.k@imim.pl (K.G.-N.); p.panek@imim.pl (P.P.); 3 Faculty of Non-Ferrous Metals, AGH University of Science and Technology, Al. Mickiewicza 30, 30-059 Kraków, Poland; 4 Hanplast, W. Paciorkiewicza 3 St., 85-862 Bydgoszcz, Poland; l.majchrowicz@hanplast.com; 5 Jerzy Haber Institute of Catalysis and Surface Chemistry, Polish Academy of Sciences, Niezapominajek 8, 30-239 Kraków, Poland

**Keywords:** substitutional doping, Burstein–Moss shift, Ge doping, transparent conducting oxides

## Abstract

In this work, we report on the fabrication of ZnO thin films doped with Ge via the ALD method. With an optimized amount of Ge doping, there was an improvement in the conductivity of the films owing to an increase in the carrier concentration. The optical properties of the films doped with Ge show improved transmittance and reduced reflectance, making them more attractive for opto-electronic applications. The band gap of the films exhibits a blue shift with Ge doping due to the Burstein–Moss effect. The variations in the band gap and the work function of ZnO depend strongly on the carrier density of the films. From the surface studies carried out using XPS, we could confirm that Ge replaces some of the Zn in the wurtzite structure. In the films containing Ge, the concentration of oxygen vacancies is also high, which is somehow related to the poor electrical properties of the films at higher Ge concentrations.

## 1. Introduction

Zinc oxide is an *n*-type semiconductor, and is one of the well-studied compounds in the family of transparent conducting oxides (TCOs). ZnO has a direct band gap (Eg) of 3.3 eV and a low exciton binding energy (60 meV) [1]. It consists of earth-abundant Zn and it is non-toxic, making it an ideal material to replace another widely used TCO, namely Indium-doped Tin oxide (ITO), which contains the scarcely available Indium. As a result of these attributes, ZnO and its variants are applied to opto-electronic devices like amorphous Si solar cells as transparent electrodes [2], in thin film CIGS/CZTS solar cells as window layers [3,4], and in perovskite solar cells as electron transporting layers [5,6]. ZnO has a wurtzite crystal structure, which is the same as GaN. This, coupled with a broadband UV emission, makes ZnO a strong candidate to replace the expensive GaN in the domain of light-emitting diodes (LED) [7,8], although much work is needed to achieve this.

Despite the interesting properties and promising potential of ZnO in several areas, intrinsic ZnO has low carrier concentrations at room temperature (typically between 10^18^ and 10^19^ cm^−3^) and, as a result, low conductivity. The magnitude of the *n*-type conductivity and the carrier concentration can be readily tuned by doping ZnO with electron-donating atoms. In this respect, the most widely known dopant for ZnO is Al. Aluminum-doped ZnO (AZO) films are very common, and AZO films improve the electrical and optical properties of ZnO considerably. However, caution should be exercised when using Al as it is expected to cause problems in ZnO. The solubility of Al in ZnO is only 0.3 mol% [9]. With excess aluminum doping, non-conductive Al_2_O_3_ clusters form in the films due to the high reactivity of Al ions, even under mild temperatures [10]. The presence of Al_2_O_3_ clusters is known to cause lattice disorders. Al_2_O_3_ is electrically insulating and can ultimately increase the electrical resistivity of the ZnO films [11]. The other elements in Group III, such as In and Ga, are scarce elements. They form an integral part in III-V devices which are indispensable in the microelectronics sector; hence, they are not feasible options when the commercialization of ZnO-based TCOs is taken into account. Group IV elements offer the same advantages as Group III elements and are earth-abundant (except Fl). Of the available elements (besides C), Si and Ge are particularly interesting due to their non-toxic nature and stable oxidation states. Although Si is extremely abundant, it has a very low ionic radius (0.26 Å), and it cannot be used as an efficient dopant. Ge, on the other hand, has an ionic radius of 0.53 Å, which is close enough to Zn (0.6 Å–0.74 Å) and could readily modify the properties of ZnO when substituted in place of Zn.

ZnO films doped with Ge have been reported earlier; they can be applied in the areas of photo electrochemical splitting, photonics, thin film transistors, and heterojunction solar cells [12,13,14,15]. This proves the versatility of the compound. The challenge, however, is to obtain optimally doped films in order to obtain the best optical/electrical properties possible. One of the best-known techniques to achieve this is by Atomic Layer Deposition (ALD). ALD is capable of depositing films with uniform thickness over large areas without the formation of defects like pin holes. ALD films can also be coated on uneven surfaces, cracks, and trench structures (conformality), which is often needed in microelectronic devices at relatively lower process temperatures [16]. Since the film is grown monolayer by monolayer in a self-limiting manner, it is easy to control the exact percentage of the dopant in the film.

In this work, Ge-doped ZnO (referred to as GZO from now on) thin films were deposited by ALD. The Ge content in the films was varied by altering the pulse ratio of the Ge and Zn precursors in the ALD cycle. The Ge content varied from 0% (undoped) to values up to 30% of the pulse ratio between Ge and Zn. The primary motivation behind this work is to study the optical/physical properties of the GZO films, exploring the bonding nature of Ge in ZnO, and understanding how the incorporation of Ge modifies the electronic structure of ZnO.

## 2. Materials and Methods

### 2.1. Fabrication of the Thin Films

#### Atomic Layer Deposition

ZnO thin films doped with Ge have been prepared using ALD equipment (Beneq P400A ALD system, Espoo, Finland) at a reactor temperature of 200 °C. The recipe for deposition is based on the work of Chalker et al. [17]. However, given the difference in the size of the reactors used in both cases, the method used in this work differs slightly in certain aspects. Prior to the start of the process, the chamber was evacuated down to a base pressure of 1 mTorr. Diethylzinc in liquid form (DEZ) (Lanxess Organometallics GmbH, Berkgamen, Germany) and Tetramethoxygermanium, (liquid, Ge(OCH_3_)_4_) (Gelest, Inc., Morrisville, NC, USA), were used as precursors for Zn and Ge, respectively. Water vapor was used as the co-reactant. The GZO films were deposited in the following method: First, a few layers of ZnO were deposited by exposing the surface to successive pulses of DEZ and then to water vapor. N_2_ purging steps (99.999% purity, PSA Nitrogen Gas Generator, Parker for 2 s) were applied in between steps to prevent gas-phase pre-reactions, to drive away intermediates from the sample surface, and to ultimately ensure a surface reaction. After the deposition of a few ZnO cycles, a single Ge-O cycle is deposited by pulsing the Ge precursor and then the water vapor. The Ge-O cycle contains one or more pulses of Ge depending on the desired Ge content. For example, if the cycle ratio of Ge to Zn is set to 5%, then one full ALD cycle or a super cycle will have the sequence of (10 × ZnO cycles + 1 × GeO cycle + 9 × ZnO cycles). This sequence was repeated many times to obtain the desired thickness. The precursor opening times of both DEZ and Ge(OCH_3_)_4_ were kept to 0.3 s, while that of water was kept to 0.5 s.

The films were deposited on two types of substrates—quartz glass, which is typically 1 mm thick ((Helma, Müllheim, Germany), and an ultra-flat single-side polished silicon wafer Si <100> (Alpha Nanotech Inc., Vancouver, BC, Canada) which is 0.18 mm thick. Before ALD, the quartz substrates were cleaned by the following procedure: The substrates were first rinsed in deionized water (DI) with 2% Hellmanex III solution (HelmaAnalytics, Müllheim, Germany) after which the solution with the substrates is ultrasonicated at 353 K for 5 min. This is followed by rinsing the substrates with DI water and isopropanol (IPA). The substrates are once again ultrasonicated in IPA at 323 K for 5 min. The cleaning is finished by rinsing the substrates for the last time in DI water where they are stored thereafter. The substrates are dried with nitrogen before use. The Si (100) wafers were used for the quantification of thickness and chemical composition, whereas the quartz substrates were used for the analysis of optical and electrical properties.

Before loading the samples in the chamber, they were surface-activated with the use of an argon plasma (Diener tetra 30, Ebhaussen, Germany). The sample chamber was pumped down to 0.3 mbar, after which the RF generator was turned on and adjusted to a power of 120 W, lasting for 2 min.

### 2.2. Film Characterization

#### 2.2.1. Scanning Electron Microscopy (SEM)

The chemical composition of the films was analyzed using scanning electron microscopy (SEM) JEOL JCM-6000 (Freising, Germany). The ratio of Ge/(Ge + Zn), which is used to quantify the amount of Ge in the films, was determined with the help of an energy-dispersive detector (EDS), which is a part of the SEM.

#### 2.2.2. Spectroscopic Ellipsometry

The nominal thickness and the refractive index of the as deposited GZO films were measured with a Sentech SE800 PV spectroscopic ellipsometer at an incident angle of 70° in the wavelength range of 280–980 nm. An optical model which consists of air/roughness/GZO/SiO_2_/Si from the surface was used for the calculations of thickness and refractive index. In order to fit the experimental data and define the optical constants of the GZO films, the Tauc–Lorentz model with four oscillators was used.

#### 2.2.3. X-ray Photoelectron Spectroscopy (XPS)

The X-ray photoelectron spectra (XPS) for the different films were recorded for the different films using a hemispherical analyzer EA 15 (PREVAC) equipped with a dual-anode X-ray source RS 40B1 (Rogów, Poland). Al Kα (1486.6 eV) radiation was used as the probing beam with an analyzer pass energy of 100 eV. The spectra were recorded in normal emission geometry with an energy resolution of 0.9 eV. The spectrometer was calibrated with the Ag, Au, and Cu foil according to the ISO 15472:2010 standard. An ultra-high vacuum (UHV) close to 8 × 10^−9^ mbar was maintained during the measurements. The sample area subjected to analysis was approximately 3 mm^2^, and the penetration depth in the films was approximately 10 nm. The samples were mounted and positioned in a dedicated holder, pumped out to high vacuum, and then transferred into the UHV chamber. The survey spectra and the high-resolution absolute spectra were acquired for all the films. The acquired spectra were analyzed with the use of CasaXPS (version 2.3.24PR) software. The electron binding energy (BE) scale was calibrated for the Fermi edge at 0.0 eV. The background was subtracted in all the data using a Shirley type spectrum. The absolute spectra for each element were analyzed by peak fitting with a Voigt function (Gauss-to-Lorentz profile ratio of 70:30). The spectra were compared with respect to the background level.

#### 2.2.4. Electrical Properties

The electrical properties of the different GZO films were measured using a ECOPIA Hall effect measurement system (HMS-5500, Anyang, Republic of Korea) at room temperature. The measurement was performed following the Van Der Pauw geometry with contact needles (gold-coated) carefully attached to the corners of a square sample, which ensures an ohmic response (straight line) between two consecutive needles.

#### 2.2.5. Optical Properties

The transmittance and reflectance of the GZO films were measured using an optical spectrophotometer (Lambda 950S, Perkin Elmer, Waltham, MA, USA).

#### 2.2.6. Kelvin Probe Force Microscopy (KPFM)

The work function of the different GZO films was measured with KPFM. First, the measurements of the contact potential difference (*CPD*) between the measuring tip and the surface of the film were carried out. Then, the work function φ of the film was calculated as follows:

$$CPD=\frac{\varphi -\varphi_{AuRE}}{e}$$

where *e* is the elementary charge, 1.6 × 10^−19^ C. The $\varphi_{Au}$ value was determined after calibration of the probe with a reference electrode (*RE*) made from HOPG (Highly Ordered Pyrolytic Graphite); for the gold probe, a value of φ = 4.815 eV was adopted.

## 3. Results and Discussion

### 3.1. Discussion on Physical and Electrical Properties

The chemical composition of the films measured by EDS is shown in Table 1. As the pulse ratio of Ge to Zn in the ALD cycle is increased, an increase in the Ge content of the GZO films is observed. The Ge content grows at the expense of Zn in the films. This suggests that Ge could substitute Zn in the host lattice. The $\frac{\left[Ge\right]}{\left[Ge\right]+\left[Zn\right]}\;$ ratio (or the GGZ ratio) ratio obtained from the EDS changes linearly with the $\frac{Ge}{Ge+Zn}\;$ pulse ratio used in the ALD reactor, as shown in Table 1. From now on, the $\frac{Ge}{Ge+Zn}\;$ pulse ratio will be simply referred to as the GGZ pulse ratio. 

The electrical properties (conductivity, carrier density, and mobility) at room temperature for the different GZO films are summarized in Table 2. The highest values of conductivity are obtained for a nominal Ge content of 6% (which corresponds to 1.8 at% of Ge). Similar observations have been previously reported for Ge-doped ZnO films [17]. Increasing the Ge content beyond this value only results in a degradation of the electrical properties, especially the conductivity and the carrier mobility. A detailed explanation of the gradation of the electrical properties as a function of the Ge content and the annealing temperature has been provided in our previous work [18]. Therefore, in this article we only provide a short summary of the physical and electrical properties.

The targeted film thickness for all the films discussed in this work is 100 nm. However, when the GGZ pulse ratio exceeds 10%, there is a drop in the thickness of the films. Similar effects on Ge-doped ZnO films have been reported earlier [17]. The decrease in the thickness of the films is due to the saturation of the growth per cycle (gpc) value which occurs at high temperatures like 200 °C. The saturation of the gpc is most likely caused by steric hindrance. In the case of pure ZnO films prepared by ALD from the same precursors, the steric hindrance has been reported to be non-existent at temperatures between 150 °C and 200 °C [19]. In the case of Ge-doped films, the reaction between the OH terminated ZnO film and the incoming Ge(OCH_3_)_4_ is catalyzed by water and promotes Ge incorporation in the ZnO films [17] according to the following equation:

(1) $$\mathrm{Ge}\;{\left({\mathrm{OCH}}_{3}\right)}_{4}+O-H:\mathrm{ZnO}\;+{\left(H_{2}O\right)}^{*}\to \;\mathrm{Ge}+4{\;\mathrm{CH}}_{3}\mathrm{OH}+\mathrm{ZnO}$$

This reaction produces CH_3_OH as a byproduct, which would dissociate in the chamber operating at 200 °C; some of these OH bonds are likely adsorbed on the surface of the film. The incoming bulky ligand species generated in successive ALD cycles, which comprise precursor intermediates from DEZ and Ge(OCH_3_)_4_, along with a growing number of hydroxyl groups on the films, offer favorable conditions for steric hindrance. Under these circumstances, the amount of Ge incorporation is likely inhibited at higher GGZ pulse ratios. From the EDS data, it can be inferred that although the concentration of Ge increases with the GGZ pulse ratio, the increase in the atomic ratio is not strictly linear. For instance, in the case of 20% Ge-doped films, the atomic percentage of Ge is about 5.5% and not 7.2% (4 × 5% GGZ pulse ratio) as one could calculate. These arguments indicate that the growth of the films per cycle is restricted to higher GGZ pulse ratios, and as a result, the desired film thickness is not obtained.

### 3.2. Discussion on Optical Prpoerties and Electronic States

**Figure 1:**
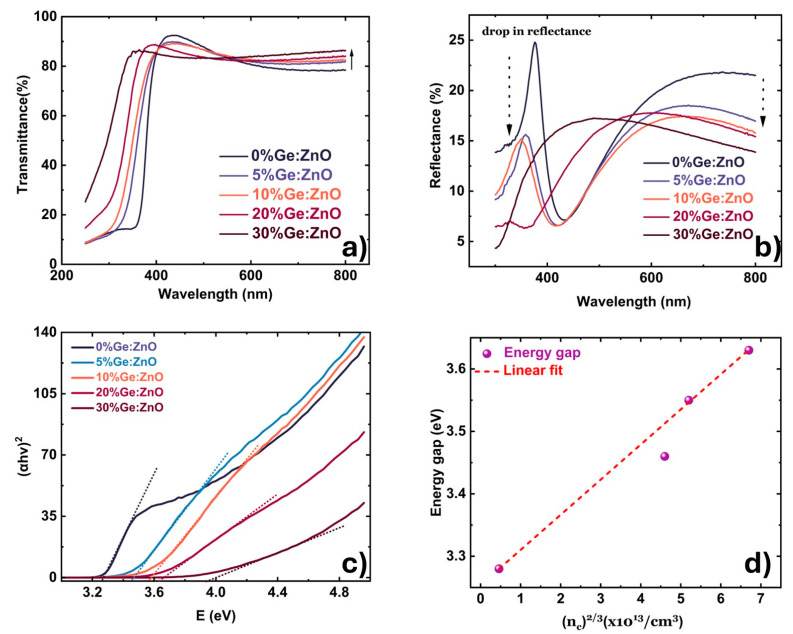
(**a**) Transmittance spectra of the different GZO films. (**b**) Reflectance spectra of the different GZO films. (**c**) Plot of (αhv)^2^ vs. hv for the various GZO thin films. The dotted line represents the linear fit to the data. (**d**) Dependence of the optical band gap and the shift in the band gap of the GZO films on the carrier density (n^2/3^). The dotted line represents a linear fit of experimental results.

The transmittance spectra of the GZO films under investigation are shown in Figure 1a. The transmittance has been measured from 250 nm to 800 nm. The average values of the transmittance in the visible region (from 550 to 800 nm) of the GZO films show an increase compared to the undoped ZnO film. The apparent increase in the transmittance of the films with increasing Ge content might indicate a reduction in the absorption of the films. Upon examining the reflectance spectra, a similar observation was made.

The reflectance of the GZO films shows a quick decline even for low amounts of Ge doping in the ZnO. The drop in the reflectance is strongly pronounced in the UV region (300 nm–420 nm, as seen in Figure 1b) for all the GZO films compared to the rest of the spectra. A decrease in the reflectance of the GZO films is also correlated with a decrease in the refractive index of the material (measured at 632 nm), which changes from 1.98 to 1.76. The optical properties of the GZO were investigated further with the calculation of the absorbance using the following equation [20]:

(2) $$\;\alpha =-\frac{1}{d}ln\left(\frac{T}{1-R}\right)$$

where *α* is the absorbance, *d* is the film thickness, and *T* and *R* are the transmittance and reflectance values of the GZO films, respectively. With the calculated values of *α*, the direct band gap of the different films was calculated from the Tauc plot, in which (αhν)^2^ is plotted against hν and the linear part of the spectrum is extrapolated to *α* = 0, as shown in Figure 1c.

The band gap of undoped ZnO is 3.26 eV. With the inclusion of Ge in the ZnO films, the band gap steadily increases. The numerical changes in the bandgap with the amount of Ge is shown Table 3. The n-type behavior and the reasonably high values of conductivity in transition metal oxides like ZnO are mainly due to the large electronegativity of oxygen [21], which originates from the delocalized conduction band’s s-like wave functions. As a result, a significant blueshift of the optical band gap is expected when the oxide is doped. Such an effect was observed for CdO, where the absorption onset was pushed above the visible range with increasing values of carrier concentration. This was accompanied by an increase in the material transparency [22], which is precisely the same scenario as our GZO films. In the current discussion, an increase in the carrier concentration of the films is observed with increasing Ge content. Likewise, there is a similar effect with the band gap. When Ge substitutes Zn in the host lattice, it offers two additional electrons, leading to a considerable increase in the carrier concentration of the Ge-doped films, as shown in Table 1. The increase in the carrier concentration can shift the Fermi level in ZnO upwards in the conduction band, resulting in band filling which is observed as an increase in the energy gap of the film. This phenomenon is called the Burstein–Moss (BM) effect [23,24].

The shift in the energy gap according to the BM effect ($E_{BM})$ is given by:

(3) $$E_{BM}=\frac{h^{2}}{8\pi^{2}m_{eh}^{*}}\;\left(\sqrt[{3}]{9\pi^{4}n_{c}^{2}}\right)$$

where *h* is the Planck constant, $m_{eh}^{*}$ is the effective electron–hole mass, and $n_{c}$ is the carrier concentration in the undoped ZnO and GZO films. In order to confirm that the energy shift is in accordance with the BM effect, the band gap and the shift in the band gap are plotted versus n^2/3^, as shown in Figure 1d. A linear relationship between the shift in the energy gap and the carrier concentration illustrates that the energy shift caused by Ge inclusion in the films is in accordance with the BM theory. From the slope of the linear fitting shown in Figure 1d, an electron–hole effective mass value of 0.4$m_{0}$ is obtained, where $m_{0}$ is the free electron mass. Theoretical calculations on ZnO have predicted values of 0.28$m_{0}$ and 0.59$m_{0}$ for the electron and hole massses in the conduction and valence bands, respectively [25,26]. Using these values, the theoretical electron–hole effective mass is calculated to be 0.19$m_{0}$. The calculated value of this effective mass from the measurements deviates from the theoretical value by a factor of two, due to the fact that the BM theory assumes parabolic dispersion of the bands. In the case of heavily doped semiconductors, this discrepancy is explained by involving the band gap renormalization (BGR) model. The BGR is a complex model which encompasses many body interactions such as electron exchange interactions, minority carrier correlations, carrier–ion interactions, and also includes the non-parabolicity of the conduction bands [27,28,29]. Band structure calculations have shown that energy shifts caused by band gap renormalization can range up to 1.1 eV for Ge-doped In_2_O_3_ when the carrier concentration reaches values up to 10^21^ cm^−3^ [30]. The dispersion of the conduction band is significantly modified when Ge is substituted into the lattice. This dispersion is caused by the hybridization of the dopant s states with the host conduction band. Upon doping, the conduction band minimum (CBM) gains a significant amount of the dopant’s character. Ge has two more electrons than Zn and, consequently, two additional protons. With an increase in the number of protons, the nuclear charge of Ge will increase, thereby increasing the nuclear force that holds the protons together. Since protons are positively charged, they repel each other due to Coulombic forces. This Coulombic repulsion will be higher in Ge than Zn, both of which have filled 4s orbitals. In order to overcome the repulsive forces and stabilize the nucleus, the nuclear binding energy of Ge has to be higher than that of Zn. This would also result in the 4s orbitals having a higher binding energy than Zn. Hence, the Ge 4s orbital lies at a higher binding energy than the Zn 4s orbital due to the increased nuclear charge. The orbital coupling leads to two effects: a lowering of the conduction band minimum, and a small decrease in the band curvature close to the band edge. These effects are at the heart of the significantly high contributions from BGR to the energy gap for Ge_Zn_ (an antisite defect, where Ge occupies the position of Zn); a decrease in the dispersion of the conduction-band leads to a much slower rise in the Fermi level upon the inclusion of carriers by doping. This is especially true when the GGZ ratio increases above 6%. Although the addition of carriers can lower the energetic positions of both the valence and conduction bands through an enhanced electron exchange potential, it is the change in the shape (i.e., the non-parabolic nature) of the bands that has the strongest effect on the shift in optical transitions [30]. The non-parabolicity of the conduction band is chiefly responsible for the significant deviations in the electron–hole effective mass between experimental and theoretical values. Large values of the electron–hole effective mass in the range of 0.59$m_{0}$–0.76$m_{0}$ from both experiements and theory have been reported in the literature [30,31].

**Figure 2:**
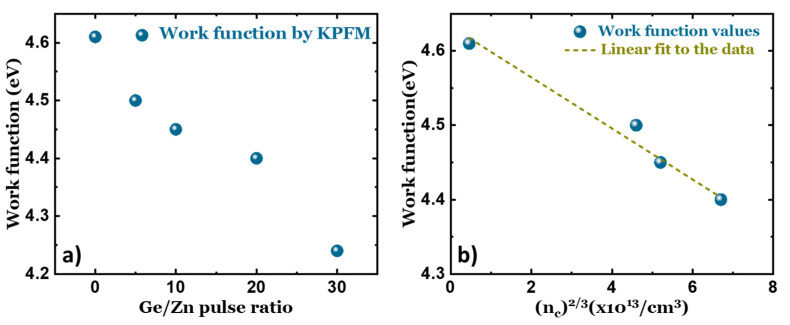
(**a**) Semiconductor work function of the various GZO films measured by KPFM from the film surface. (**b**) The dependence of the work function on the carrier density (n^2/3^) of the films. The dotted line represents a linear fit of experimental results.

In order to shed more light on the change in the electronic states of ZnO caused by Ge doping, the work function was measured. The work function values measured for the different Ge-doped films are presented in Figure 2a. From the measurements, the work function of undoped ZnO was found to be 4.61 eV; moreover, with an increase in the GGZ ratio, the value of the work function is reduced systematically, as observed in Figure 2a.

In order to establish a theoretical understanding of the work function, a simplified model where the influence of the surface states is not taken into account is proposed. In such cases, the work function of a degenerately doped ZnO film can be expressed as follows:

(4) $$\;\varphi =E_{vac}-E_{C}+E_{F}$$

where $E_{vac}$ is the electron energy at vacuum level, which is typically 0 eV, $E_{C}$ is the energy of the electron at the bottom of the conduction band, and $E_{F}$ is the Fermi level. In terms of the effective electron mass, the Fermi level can be given by the following expression:

(5) $$E_{F}=\frac{h^{2}}{8\pi^{2}m_{c}}\;K_{F}^{2}\;$$

where $m_{c}$ is the effective mass of the electron in the conduction band and $K_{F}$ is the Fermi wave vector at the Fermi energy up to which all the states are filled under the assumption of a free electron model. The magnitude of $K_{F}$ is given by the following expression:

(6) $$K_{F}={\left(3\pi^{2}n_{c}\right)}^{1/3}$$

When this value is substituted in Equation (4), we observe that the Fermi level can be expressed as a function of the carrier concentration. When the work function is plotted against ($n_{c}$)^2/3^, as shown in Figure 2b, it shows a negative linear relationship with the carrier concentration of the films, a trend opposite to that of the band gap. This indicates that upon increasing the Ge content in the films, the carrier density also increases, leading to the empty states in the conduction band being filled. As the band states are being occupied continuously, the Fermi level is shifted into the conduction band, causing a reduction in the measured work function, according to Equation (4). The slope of the obtained linear fitting yields an effective mass of 0.67$m_{0}$ after calculations. Jia et al. reported an electron-effective mass of 0.95$m_{0}$ when Al was doped into sputtered ZnO films [28]. Comparing this with the predicted theoretical electron mass, which is 0.28$m_{0}$, there is a considerable deviation. The main causes behind this difference could arise from the segregation of impurities (secondary phases) in the film and/or the non-parabolic nature of the conduction band, which was discussed in detail previously. The presence of impurity-like features in the film has been shown to affect the work function of ZnO very strongly [32,33,34]. A more detailed discussion on the presence of secondary phases and surface states is provided in the upcoming section.

### 3.3. Discussion on the Surface States

The surface composition and the nature of the chemical bonding in the GZO films were analyzed by XPS. From the XPS measurements, the deconvoluted absolute spectra were measured. The absolute spectra contain information on the core binding energies of the elements, which in turn reveal the chemical surroundings of the individual atoms. The peak assignments which correspond to different chemical states of the atoms were carried out based on the Handbook of X-ray Photoelectron Spectroscopy and the X-ray Photoelectron Spectroscopy Database [35,36].

**Figure 3:**
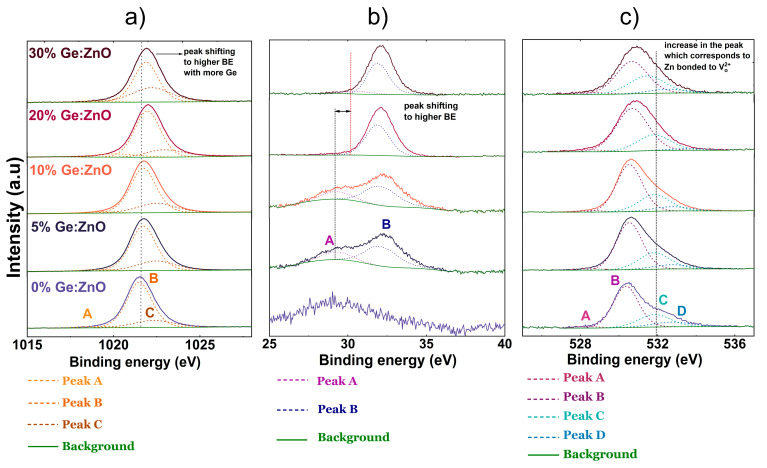
(**a**) The deconvoluted absolute spectra of the Zn 2p state (only the intense 2p_3/2_ peak is shown for clarity and simplicity) for the different GZO films. (**b**) The deconvoluted absolute spectra of the Ge 3d state (only the intense 3d_5/2_ peak is shown for clarity and simplicity) for the different GZO films. (**c**) The deconvoluted absolute spectra of the O 1s state for the different GZO films.

The XPS analysis of the absolute spectra of the Zn 2p line is shown in Figure 3a. Zn 2p has a doublet, namely Zn 2p_1/2_ and Zn 2p_3/2_, due to spin–orbit coupling. Since the doublets have identical features and differ only in the ratio of the areas, the upcoming discussion is limited to only the intense peak which corresponds to the Zn 2p_3/2_ state. The intense peak which lies between 1021.5 and 1022 eV (Table 4) in all the films can be deconvoluted into three components as shown in Figure 3a. The peak with the lowest BE of 1019 eV is present in all the samples and in almost the same proportion. This peak corresponds to the bonding of Zn with carbon species, the origin of which is suspected to be that of adventitious carbon, which is present in almost all XPS analysis. Another possibility that can explain the origin of this peak is the residual traces of the organic precursor arising from one of the ALD half reactions. The most intense component among the three, which is the peak at 1021.8 eV, corresponds to the bonding of Zn with O in the ZnO lattice. The percentage area of this peak is shown in Figure 3a, from which it is very clear that as the GGZ pulse ratio is increased, the peak area is decreased. This trend aligns with the observations from the SEM-EDX measurements and supports the observation that Zn is being substituted by Ge in the host lattice. Additionally, we also notice that the position of this peak is shifted to a higher binding energy (BE) with the inclusion of Ge in the films. The reason behind this shift in BE is due to the difference in the electronegativities of the concerned atoms and the difference in the oxidation states. Ge has a higher electronegativity than Zn, meaning the electron density around Ge bonded to O is lowered; hence, the binding energy increases. On the other hand, Ge has an oxidation state of 4+, higher than Zn^2+^, which also contributes to the chemical shift. The peak at the highest BE of about 1022 eV corresponds to Zn bonded with OH groups. ZnO terminated by hydroxyl groups at the surface is a well-known and widely reported aspect [37]. However, it is interesting to note that the proportion of this peak grows with the increasing Ge content in the films.

When we look at the Ge 3d absolute spectra shown in Figure 3b, we notice doublets which correspond to the Ge 3d_3/2_ and Ge 3d_5/2_ states, respectively; for the discussion, we will consider the Ge 3d_5/2_ emission peak. The main peak can be deconvoluted into two components. The smaller of the two peaks, which starts at 29.2 eV for the 5% film and ends at 30.5 eV for the 30% film, can be assigned to interactions between Ge bonded to C or Ge bonded to O in the lattice which contains O vacancies which are present in ZnO. The magnitude of the shift indicates a change in the bonding environment of Ge, which increases considerably with the amount of Ge incorporated in the film. The nature of this shift suggests that it is highly unlikely for the peak at 29.2 eV to be related to Ge bonded with adventitious carbon or C originating from the Ge precursor. The larger of the two peaks at around 32 eV is assigned to the bonding of Ge with O present in the wurtzite structure. However, the BE of this peak is lower by 1 eV compared to the Ge-O bond in stoichiometric GeO_2_ and higher by 3 eV than elemental Ge, both of which correspond to an oxidation state of 4+, which is the same as the substituted Ge in ZnO. The difference in the BE for the same oxidation state suggests that the electronic environment around Ge present in ZnO is very different from that of stoichiometric GeO_2_, wherein the Ge in the ZnO lattice is bonded to O and/or surrounded by O vacancies which lower the electronic interaction with Ge. The proportion of this peak increases with the GGZ ratio as expected, implying that with more available Ge, the extent of substitution in the ZnO lattice increases. 

**Figure 4:**
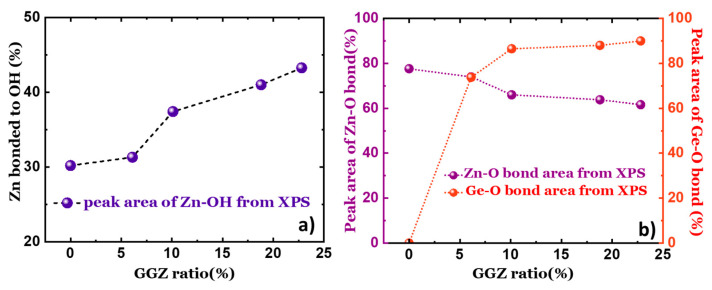
(**a**) The peak area of Zn-O bonds deduced from the deconvolution of the Zn 2p_3/2_ spectra and the peak area of Ge-O bonds deduced from the deconvolution of the Ge 3d_3/2_ spectra plotted as a function of the GGZ ratio. (**b**) The peak area corresponding to the bond of Zn-OH deduced from the deconvolution of the Zn 2p_3/2_ spectra and O 1s spectra plotted as a function of the GGZ ratio.

The detailed features of the O 1s peak are shown in Figure 3c. The peak can be fitted with four Voigt functions. The peak with the lowest BE of the four, the one centered at 528 eV, is present in all the films and its proportion varies only slightly in the four films. This peak can be attributed to the presence of C or H bonded to O on the surface. The next highest BE at about 530.5 eV is assigned to bonding with Zn from the oxide lattice without O vacancies, whilst the BE at 531.8 eV can be assigned to Zn bonding with O in the oxide lattice with O vacancies. The peak with the highest BE centered at 532.8 eV is associated with Zn bonded to O from hydroxyl groups on the surface [38,39]. The peak area corresponding to the bonding of Zn with OH calculated from the Zn 2p_3/2_ peak and O 1s were combined and plotted as a function of the GGZ ratio. A linear relationship is observed between the amount of Zn-OH bonds and the GGZ ratio (shown later in Figure 4a). This is due to the fact that with an increasing amount of Ge pulses in the ALD cycle, more OH groups are created in the reaction according to Equation (1), where higher GGZ pulse ratios result in steric hindrance.

The peak areas which represent bonding of Zn with vacancies increase with an increase in the GGZ ratio. Similar phenomena have been reported earlier in ZnO films [40,41]. The amount of O vacancies in ZnO has been shown to increase when ZnO is doped with cations such as Cr^3+^, Mn^2+^, and Co^2+^ due to the dopant-induced charge transfer mechanism. This charge transfer is strongly pronounced in ZnO when the host atom and the substituting atom have different oxidation states. When an oxygen atom with a neutral charge is displaced from its actual site in the lattice it results in the formation of a vacancy along with the generation of two extra charges. This is shown in Equation (7) using the Kröger Vink notation [39]. The creation of oxygen vacancies at higher GGZ pulse ratios takes place according to Equation (8):

(7) $$O_{0\;}^{x}\;⇌{\;V}_{0}^{{}^{\circ}{}^{\circ}}+2e^{\prime }$$

(8) $$\mathrm{ZnO}+{\mathrm{Ge}}^{4+}\;\to \;\mathrm{GeZn}0_{1-x}+\mathrm{Ge}-O_{x}\;\mathrm{where}\;x<1$$

The purpose of Equation (8) is only to show that O vacancies could be created in this way at higher GGZ ratios. This process also results in the formation of microscopic Ge-O clusters. These Ge-O clusters are reported to act as carrier traps and not as electron donors [42,43]. As a result, the electrical properties of the Ge-doped films with increasing Ge content show a degradation. The presence of Ge clusters could not be detected from Grazing Incidence X-ray Diffraction (GI-XRD) measurements which were carried out in our previous work [18]. This is possibly due to two reasons: one, these clusters are present in very small amounts in the bulk, and hence, are insensitive to surface measurements. Second, these clusters have short-range ordering and do not really exhibit a diffraction pattern when probed by X-rays. Nevertheless, the presence of these clusters cannot be discarded. The Ge-O clusters could result in the segregation of impurities in the bulk of the film. In the previous section, a significant deviation in the electron effective mass between theoretical and experimentally measured values was shown. Along with the non-parabolic conduction bands, the presence of such impurity phases can also influence the optical properties of ZnO with the increased electron effective mass being one such manifestation.

The peak areas corresponding to the bonding of Zn with OH calculated from the Zn 2p_3/2_ peak and the O 1s peak were combined and plotted as a function of the GGZ ratio as shown in Figure 4a. A linear relationship is observed between the number of Zn-OH bonds and the GGZ ratio. This is due to the fact that with an increasing amount of Ge pulses in the ALD cycle, more OH groups are created in the reaction, according to Equation (1), where higher GGZ pulse ratios result in steric hindrance. Similarly, the peak areas that correspond to the Zn-O bond and the Ge-O bond were plotted on a double Y scale as a function of the GGZ ratio, as shown in Figure 4b. A decrease in the peak area of the Zn-O bonds is accompanied by an increase in the peak area of Ge-O bonds simultaneously. This trend confirms substitutional doping by Ge at the sites occupied by Zn in the wurtzite structure.

## 4. Conclusions

A series of Ge-doped ZnO thin films have been successfully deposited by ALD in a controlled manner. The amount of Ge incorporated in the ZnO films increases with an increase in the Ge/Zn pulse ratio for all the values discussed in this work. However, for higher GGZ pulse ratios (>10%), there is a saturation in the gpc which leads to a reduction in the thickness of the films. The electrical properties of the films show an improvement for Ge concentrations up to 1.8 at. percentage, beyond which there is a degradation. However, even for very high values of Ge incorporated in the film, the optical properties (T and R) are improved. The band gap of the films is blue-shifted with increasing values of Ge in the film. The band gap and the work functions of the films exhibit a strong dependance on the carrier concentration of the films which, when exceeding a threshold value, shifts the Fermi level into the conduction band of ZnO. With the help of detailed XPS analysis, substitutional doping of Zn by Ge could be verified. The fact that the work function and the band gap can be tuned precisely and effectively by Ge doping offers interesting possibilities and makes ZnO a promising candidate for applications that require TCOs.

## Figures and Tables

**Table 1:** Chemical composition of the GZO films deposited on a Si wafer.

$\frac{Ge}{Ge+Zn}$ Pulse Ratio (%)	Chemical Composition (at. %)			$\frac{\left[Ge\right]}{\left[Ge\right]+[Zn]}$ Atomic Ratio (%)
	O	Zn	Ge	
0	69.9 ± 0.8	30.1 ± 0.8	-	-
5	70.4 ± 0.8	27.8 ± 0.8	1.8 ± 0.2	6.1
10	69.4 ± 1.3	27.5 ± 1.5	3.1 ± 0.5	10.1
20	70.8 ± 0.8	23.7 ± 0.6	5.5 ± 0.4	18.8
30	70.1 ± 0.6	23.0 ± 0.1	6.8 ± 0.4	22.8

**Table 2:** Electrical properties of the GZO films deposited on a quartz substrate with their thicknesses.

$\frac{Ge}{Ge+Zn}\;$ Pulse Ratio (%)	Electrical Properties			Thickness (nm)	Refractive Index (n)
	Conductivity (×10^3^ S·m)	Carrier Density (10^20^/cm^3^)	Mobility (cm^2^/Vs)		
0	9.5	0.1	20	97	1.98
5	37	3.1	7	97.3	1.92
10	22	3.7	3.8	99.4	1.86
20	2.2	5.5	0.75	86.2	1.82
30	-	-	-	65	1.76

**Table 3:** Band gap energy calculated from UV–Vis measurement.

Sample	0% Ge:ZnO	5% Ge:ZnO	10% Ge:ZnO	20% Ge:ZnO	30% Ge:ZnO
**Band gap**	3.26 eV	3.45 eV	3.60 eV	3.65 eV	3.82 eV

**Table 4:** Peak energies of the constituent elements along with their proportions, calculated after fitting the absolute spectra measured by XPS data.

Sample	Zn 2p			Ge 3d		O 1s			
	A (eV)	B (eV)	C (eV)	A(eV)	B(eV)	A (eV)	B (eV)	C (eV)	D (eV)
30% Ge:ZnO	1019.2 3.1%	1021.5 61.69	1022.5 35.3%	30.6 10%	32.2 90%	528.8 3.5%	530.8 54.4%	532.1 34.1%	532.9 8%
20% Ge:ZnO	1019.2 3.2%	1021.8 63.8%	1022.5 33%	30.3 12.2%	32.1 88.8%	528.7 3.1%	530.7 55.7%	531.7 33.6%	532.8 7.5%
10% Ge:ZnO	1019.1 3.1%	1021.7 66.7%	1022.4 30.2%	29.8 14.4%	32.1 85.6%	528.6 2.6%	530.6 56.1%	531.6 33%	532.8 6.9%
5% Ge:ZnO	1019.1 2.9%	1021.5 73.7%	1022.3 23.4%	29.3 26.2%	32.0 73.8%	528.5 2.3%	530.5 62.6%	531.8 26.5%	532.7 8.2%
0% Ge:ZnO	1018.8 3.2%	1021.5 77.7%	1022.2 19.1%	-	-	528.3 2.5%	530.4 63.2%	531.7 23.8%	532.7 11.1%

## Data Availability

Data are contained within the article.
